# Joint Effects of Febrile Acute Infection and an Interferon-γ Polymorphism on Breast Cancer Risk

**DOI:** 10.1371/journal.pone.0037275

**Published:** 2012-05-18

**Authors:** Yi Su, Lu-Ying Tang, Li-Juan Chen, Jian-Rong He, Feng-Xi Su, Ying Lin, Wei-Qing Chen, Xiao-Ming Xie, Ze-Fang Ren

**Affiliations:** 1 The School of Public Health, Sun Yat-Sen University, Guangzhou, China; 2 The Third Affiliated Hospital, Sun Yat-Sen University, Guangzhou, China; 3 The Second Affiliated Hospital, Sun Yat-Sen University, Guangzhou, China; 4 The First Affiliated Hospital, Sun Yat-Sen University, Guangzhou, China; 5 Department of Breast Oncology, Sun Yat-Sen University, Guangzhou, China; University of Medicine and Dentistry of New Jersey, United States of America

## Abstract

**Background:**

There is an inverse relationship between febrile infection and the risk of malignancies. Interferon gamma (IFN-γ) plays an important role in fever induction and its expression increases with incubation at fever-range temperatures. Therefore, the genetic polymorphism of *IFN-γ* may modify the association of febrile infection with breast cancer risk.

**Methodology and Principal Findings:**

Information on potential breast cancer risk factors, history of fever during the last 10 years, and blood specimens were collected from 839 incident breast cancer cases and 863 age-matched controls between October 2008 and June 2010 in Guangzhou, China. *IFN-γ* (rs2069705) was genotyped using a matrix-assisted laser desorption/ionization time-of-flight mass spectrometry platform. Odds ratios (OR) and 95% confidence intervals (CIs) were calculated using multivariate logistic regression. We found that women who had experienced ≥1 fever per year had a decreased risk of breast cancer [ORs and 95% CI: 0.77 (0.61–0.99)] compared to those with less than one fever a year. This association only occurred in women with CT/TT genotypes [0.54 (0.37–0.77)] but not in those with the CC genotype [1.09 (0.77–1.55)]. The association of *IFN-γ* rs2069705 with the risk of breast cancer was not significant among all participants, while the CT/TT genotypes were significantly related to an elevated risk of breast cancer [1.32 (1.03–1.70)] among the women with <1 fever per year and to a reduced risk of breast cancer [0.63 (0.40–0.99)] among women with ≥1 fever per year compared to the CC genotype. A marked interaction between fever frequencies and the *IFN-γ* genotypes was observed (P for multiplicative and additive interactions were 0.005 and 0.058, respectively).

**Conclusions:**

Our findings indicate a possible link between febrile acute infection and a decreased risk of breast cancer, and this association was modified by *IFN-γ* rs2069705.

## Introduction

Since the 19^th^ century, it has been repeatedly observed that spontaneous cancer regressions were coincided with acute infections and the cancer patients had a remarkable disease-free history before the onset of cancer [1]–[4]. In the 20^th^ century, an inverse association between infectious diseases, particularly febrile ones, and cancer risk has also been consistently found for malignant melanoma and glioma using modern epidemiological methods [1], [3], [5]–[7].

With the widespread introduction of antibiotics and antipyretics since the beginning of the last century, however, the critical role played by fever has often been overlooked, resulting in considerable changes to the clinical course and magnitude of the immune response that develops following acute infections [1], [8]. These changes may be part of the reasons for the substantial increase in the age-adjusted incidences or mortalities of malignant diseases during the early part of the last century in western countries [9] and in the late of the last century in China [10]. It has been observed that every 2% reduction in infectious disease mortality was followed by a 2% increase in cancer mortality over a 10-year interval from 1895 to 1963 in Italy [9]. Conventional cancer treatments have made slow progress and cancer is still an incurable ailment. Under these circumstances, there are important implications for the re-exploration of this hidden treasure buried in time—that fever induced by acute infection may be antagonistic to cancer development [2], [4]. We may take the advantage of fever, the beneficial aspect of acute infections, to prevent and treat cancer because we are able to induce and control fever much better than before [4].

Increased body temperature has been shown to stimulate the immune system through the augmentation of T and NK cell activity and the production of cytokines, including interferon gamma (IFN-γ) [11]–[13]. The IFN-γ expression has been found to increase along with the artificial elevation of fever-range temperatures in numerous studies [14]–[18]. IFN-γ has also been shown to increase after feverish infection [13]. In turn, IFN-γ is able to induce fever and can be considered an independent endogenous pyrogen in humans and rabbits [19]–[22]. Fever has been shown to be a constant side effect encountered with IFN-γ treatments [23], [24]. Moreover, a high level of IFN-γ expression shows antitumor activity [25] and IFN-γ inhibits the growth of many cell lines that originate from tumors, including breast cancer [26]. In addition, a potentially functional single nucleotide polymorphism (SNP, rs2069705) in *IFN-γ* (−1615 C/T) [27], [28] has been reported to be associated with breast cancer risk [29]. Therefore, there may be interplay between febrile infection and *IFN-γ* in relation to breast cancer risk.

In the present study, we evaluated the association of febrile acute infection with breast cancer risk, and further investigated whether this association is modified by *IFN-γ* polymorphism (rs2069705) in a case-control study in Guangzhou, China.

## Results

### Fever frequencies and genotypes in cases and controls

Breast cancer cases, compared to similarly aged controls, were more likely to be premenopausal and low educated. They were comparable in terms of marital status, body mass index (BMI), age at menarche, parity, and family history of breast cancer (Table 1). The proportions for different fever frequencies of below once, 1∼2 times, 3∼4 times, and 5 times or above per year were 76.1%, 19.3%, 3.3%, and 1.4% for patients and 72.1%, 23.0%, 3.3% and 1.5% for controls, respectively.

**Table 1:** Characteristics of breast cancer cases and controls.

Characteristic	Cases, *n* (%)	Controls, *n* (%)	*P* value
Age			
≤40	210 (25.0)	226 (26.2)	
41∼59	494 (58.9)	491 (56.9)	
60∼	135 (16.1)	146 (16.9)	0.709
Mean ± SD	48.97±11.57	49.23±11.76	0.654^b^
Education			
Junior middle school or below	389 (46.4)	315 (36.5)	
Senior middle school	216 (25.7)	326 (37.8)	
College or above	174 (20.7)	188 (21.8)	**<0.001**
Unknown	60 (7.2)	34 (3.9)	
Marital status			
Never married	41 (4.9)	33 (3.8)	
Married/living as married	711 (84.7)	717 (83.1)	
Separated/widow	46 (5.5)	63 (7.3)	0.182
Unknown	41 (4.9)	50 (5.8)	
Body mass index (kg/m^2^)			
<22	349 (41.6)	338 (39.2)	
22∼24	240 (28.6)	273 (31.6)	
≥25	206 (24.6)	196 (22.7)	0.293
Unknown	44 (5.2)	56 (6.5)	
Age at menarche (years)			
≤12	109 (13.0)	(15.5)	
>12	682 (81.3)	689 (79.8)	0.160
Unknown	48 (5.7)	40 (4.6)	
Menopausal status			
Premenopausal	479 (57.1)	402 (46.6)	
Postmenopausal	338 (40.3)	430 (49.8)	**<0.001**
Unknown	22 (2.6)	31 (3.6)	
Age at menopause (years)^a^			
≤45	72 (21.3)	72 (16.7)	
46∼50	(38.5)	182 (42.3)	
>50	99 (29.3)	155 (36.1)	0.096
Unknown	37 (11.0)	21 (4.9)	
Parity			
0	73 (8.7)	59 (6.8)	
≥1	732 (87.3)	772 (89.5)	
Unknown	34 (4.1)	32 (3.7)	0.144
Family history of breast cancer			
Absent	780 (93.0)	800 (92.7)	
Present	25 (3.0)	28 (3.2)	0.753
Unknown	34 (4.1)	35 (4.1)	

a Postmenopausal women only.

b Student's t-test.

*P* values in bold indicate statistical significance.

After adjustment for potential breast cancer risk factors, women who had experienced 1∼2 fevers per year and 3 fevers or more per year exhibited a significant and a non-significant decreased risk of breast cancer compared to those who experienced below one fever a year with ORs and 95% CIs of 0.77 (0.59–1.00) and 0.79 (0.47–1.32), respectively (Table 2). The non-significant decrease in breast cancer risk was probably the result of the small sample size at this level (only 34 cases and 38 controls). Given that the direction of the two ORs is the same, we combined these two levels. The ORs and 95% CIs for the combined level of fever frequency (≥1 times per year) were 0.77 (0.61–0.99) in the multivariate model (Table 2). The genotypes of *IFN-γ* rs2069705 were equally distributed in cases and controls and, consequently, were not significantly associated with breast cancer risk (Table 2).

**Table 2:** Multivariate odds ratio of breast cancer risk associated with previous fever frequency and *IFN-γ* genotypes.

Variables	Cases, n (%)	Controls, n (%)	OR (95%CI)^a^	OR (95%CI)^b^
Fever frequency (/year)				
<1 time	556 (76.1)	560 (72.1)	1.00 (reference)	1.00 (reference)
1∼2 times	141 (19.3)	179 (23.0)	0.79 (0.62–1.02)	**0.77 (0.59–1.00)**
≥3 times	34 (4.7)	38 (4.9)	0.90 (0.56–1.45)	0.79 (0.47–1.32)
*P* for trend			0.136	0.056
≥1 time	175 (23.9)	217 (27.9)	0.81 (0.64–1.02)	**0.77 (0.61–0.99)**
*IFN-γ* rs2069705				
CC	407 (50.4)	442 (52.6)	1.00 (reference)	1.00 (reference)
CT	332 (41.1)	342 (40.7)	1.05 (0.86–1.29)	1.06 (0.86–1.31)
TT	68 (8.4)	56 (6.7)	1.32 (0.90–1.93)	1.28 (0.87–1.90)
*P* for trend			0.204	0.245
CT+TT	400 (49.6)	398 (47.4)	1.09 (0.90–1.32)	1.09 (0.89–1.34)

a Adjusted for age.

b Adjusted for age, BMI, age at menarche, marital status, education, parity, menopausal status, and family history of breast cancer.

Values in bold indicate statistical significance.

### Associations of fever frequency and *IFN-γ* genotypes with breast cancer risk in subgroups of clinicopathological features

Subsequently, we attempted to examine whether the disease process per se or clinical characteristics affect the association of the genotypes and fever frequencies with breast cancer risk by carrying out several exploratory stratified analyses. Similar associations between breast cancer risk and fever frequency were observed across menopausal, clinical stage, and HER2 statuses (Table 3). Although the reduced risk was slightly more evident among women with BMI≥24 (OR = 0.53, 95% CI = 0.33–0.84) and patients with ER negative (OR = 0.67, 95% CI = 0.45–1.00) or with PR negative (OR = 0.66, 95% CI = 0.46–0.94), the P values for interaction or heterogeneity were not significant (Table 3). Meanwhile, the *IFN-γ* genotypes were not significantly associated with the risk of breast cancer in each stratum and no interaction between them on breast cancer risk occurred (Table 3).

**Table 3:** Multivariate odds ratio of breast cancer associated with previous fever frequency stratified by clinicopathological characteristics.

Characteristics	Fever frequency				*IFN-γ* rs2069705			
	Frequency (time/year)	Casesn (%)	Controlsn (%)	OR (95%CI)^a^	Genotype	Cases n (%)	Controls n (%)	OR (95%CI)^a^
Menopause								
Premenopausal	<1	330 (76.6)	268 (73.8)	1.00 (reference)	CC	242 (52.7)	207 (52.5)	1.00 (reference)
	≥1	101 (23.4)	95 (26.2)	0.82 (0.58–1.15)	CT/TT	217 (47.3)	187 (47.5)	0.96 (0.73–1.28)
Postmenopausal	<1	224 (75.4)	278 (69.8)	1.00 (reference)	CC	156 (47.9)	219 (52.8)	1.00 (reference)
	≥1	73 (24.6)	120 (30.2)	0.71 (0.50–1.03)	CT/TT	170 (52.1)	196 (47.2)	1.18 (0.86–1.61)
*P* a for interaction (multiplicative/additive)				0.685/0.596				0.491/0.750
BMI								
<24	<1	341 (73.5)	349 (70.9)	1.00 (reference)	CC	261 (52.2)	277 (53.0)	1.00 (reference)
	≥1	123 (26.5)	143 (29.1)	0.88 (0.65–1.18)	CT/TT	239 (47.8)	246 (47.0)	0.97 (0.75–1.26)
≥24	<1	198 (81.1)	180 (72.6)	1.00 (reference)	CC	123 (46.4)	135 (51.7)	1.00 (reference)
	≥1	46 (18.9)	68 (27.4)	**0.53 (0.33–0.84)**	CT/TT	142 (53.6)	126 (48.3)	1.24 (0.86–1.78)
*P* a for interaction (multiplicative/additive)				0.099/0.057				0.293/0.148
Clinical stage								
Localized	<1	306 (75.2)	560 (72.1)	1.00 (reference)	CC	211 (47.7)	442 (52.6)	1.00 (reference)
	≥1	101 (24.8)	217 (27.9)	0.83 (0.62–1.11)	CT/TT	231 (52.3)	398 (47.4)	1.18 (0.93–1.49)
Regional/distant	<1	207 (76.7)	560 (72.1)	1.00 (reference)	CC	158 (52.7)	442 (52.6)	1.00 (reference)
	≥1	63 (23.3)	217 (27.9)	0.75 (0.53–1.05)	CT/TT	142 (47.3)	398 (47.4)	0.99 (0.75–1.32)
*P* a for heterogeneity test				0.469				0.184
ER								
Positive	<1	367 (75.1)	560 (72.1)	1.00 (reference)	CC	273 (51.0)	442 (52.6)	1.00 (reference)
	≥1	122 (24.9)	217 (27.9)	0.83 (0.63–1.08)	CT/TT	262 (49.0)	398 (47.4)	1.05 (0.84–1.32)
Negative	<1	150 (79.4)	560 (72.1)	1.00 (reference)	CC	107 (50.2)	442 (52.6)	1.00 (reference)
	≥1	39 (20.6)	217 (27.9)	**0.67 (0.45–1.00)**	CT/TT	106 (49.8)	398 (47.4)	1.08 (0.79–1.48)
*P* a for heterogeneity test				0.235				0.911
PR								
Positive	<1	317 (74.8)	560 (72.1)	1.00 (reference)	CC	247 (52.2)	442 (52.6)	1.00 (reference)
	≥1	107 (25.2)	217 (27.9)	0.86 (0.65–1.14)	CT/TT	226 (47.8)	398 (47.4)	1.01 (0.80–1.28)
Negative	<1	199 (78.7)	560 (72.1)	1.00 (reference)	CC	133 (48.5)	442 (52.6)	1.00 (reference)
	≥1	54 (21.3)	217 (27.9)	**0.66 (0.46–0.94)**	CT/TT	141 (51.5)	398 (47.4)	1.15 (0.86–1.53)
*P* a for heterogeneity test				0.123				0.459
HER2								
Positive/equivocal	<1	153 (75.7)	560 (72.1)	1.00 (reference)	CC	115 (50.2)	442 (52.6)	1.00 (reference)
	≥1	49 (24.3)	217 (27.9)	0.82 (0.56–1.19)	CT/TT	114 (49.8)	398 (47.4)	1.09 (0.80–1.48)
Negative	<1	354 (76.5)	560 (72.1)	1.00 (reference)	CC	258 (51.3)	442 (52.6)	1.00 (reference)
	≥1	109 (23.5)	217 (27.9)	0.78 (0.59–1.03)	CT/TT	245 (48.7)	398 (47.4)	1.03 (0.82–1.30)
*P* a for heterogeneity test				0.969				0.682

a Adjusted for age, BMI, age at menarche, marital status, education, parity, menopausal status, and family history of breast cancer (excluded the stratified factor in each stratum).

Values in bold indicate statistical significance.

### Joint effects of fever frequency and *IFN-γ* genotypes on breast cancer risk

Finally, the joint effects of fever frequency and IFN-γ genotypes on breast cancer risk were analyzed. Compared to women who had experienced a fever below once a year, the reduced risk of one fever or above per year was more evident among women with the CT/TT genotypes than among those with the CC genotype with ORs and 95% CIs of 0.54 (0.37–0.77) and 1.09 (0.77–1.55), respectively (Table 4). Compared to women with the CC genotype, the risk of breast cancer in women with the CT/TT genotypes was significantly elevated among women with <1 fever per year, whereas it was significantly reduced among women with ≥1 fever per year. The ORs and 95% CIs were 1.32 (1.03–1.70) and 0.63 (0.40–0.99), respectively (Table 4). A marked interaction between fever frequencies and the *IFN-γ* genotypes on breast cancer risk was observed (P for multiplicative and additive interaction were 0.005 and 0.058, respectively).

**Table 4:** Multivariate odds ratio of breast cancer associated with previous fever frequency by genotype of *IFN-γ* rs2069705.

Variables	Variables	Cases, n (%)	Controls, n (%)	OR (95%CI)^a^	OR (95%CI)^b^
Genotype *IFN-γ* rs2069705	Fever frequency (/year)				
CC	<1 time	255 (72.9)	297 (73.7)	1.00 (reference)	1.00 (reference)
	≥1 times	95 (27.1)	106 (26.3)	1.05 (0.76–1.45)	1.09 (0.77–1.55)
					
CT/TT	<1 time	279 (79.5)	248 (69.9)	1.00 (reference)	1.00 (reference)
	≥1 times	72 (20.5)	107 (30.1)	**0.60 (0.42–0.84)**	**0.54 (0.37–0.77)**
Fever frequency (/year)	Genotype *IFN-γ* rs2069705				
<1 time	CC	255 (46.2)	297 (53.8)	1.00 (reference)	1.00 (reference)
	CT/TT	279 (52.9)	248 (47.1)	**1.31 (1.03–1.67)**	**1.32 (1.03–1.70)**
≥1 times	CC	95 (47.3)	106 (52.7)	1.00 (reference)	1.00 (reference)
	CT/TT	72 (40.2)	107 (59.8)	0.75 (0.50–1.12)	**0.63 (0.40–0.99)**
*P* b for interaction (multiplicative/additive)				**0.020**/0.055	**0.005**/0.058

a Adjusted for age.

b Adjusted for age, BMI, age at menarche, marital status, education, parity, menopausal status, and family history of breast cancer.

Values in bold indicate statistical significance.

## Discussion

Acute infections are often accompanied by inflammatory reactions including fever, which is a cytokine-mediated rise in core temperature, and other immunologic, endocrinologic, neurologic, and physiologic changes [1], [30]. A fever can establish a cascade of host defense mechanisms by inducing the proliferation and differentiation of leucocytes and the secretion of interferons (IFNs), tumor necrosis factor (TNF)-α, antibodies, and neutrophil migration [11], [12]. It has also been shown both *in vitro* and *in viv*o that dendritic cells (DC, key antigen-presenting cells in the immune system) treated with fever-like heat (41°C) were significantly superior compared to non-heat-treated DC in stimulating T cells and activating NF-κB (a transcription factor that regulates various immunological genes and plays an important role in human tumor suppression) [5], [15]. Moreover, tumor cells are more vulnerable to heat than normal cells and undergo necrosis to a larger extent [31], [32] and hyperthermia has become an auxiliary approach in cancer therapy [14], [33]–[36]. In addition, several epidemiological studies have consistently found an inverse association between febrile acute infections and cancer risk [1], [3], [5]–[7], [37]–[39], especially for malignant melanoma, which have been shown a link to breast cancer in epidemiologic and genetic studies [40]–[42]. Therefore, our finding of an inverse association between fever frequency and breast cancer risk is in agreement with the results of previous studies.

IFN-γ is one of the main products of Th1-specific proinflammatory cytokines and has an effect on host defense and immune regulation, such as antivirus, antimicrobe, and antitumor activities [43]. It has also been shown to have a high correlation to tumor regression in immunotherapy, but these results were mixed as to the efficacy in clinical trials [43]. Several studies have examined the association of polymorphisms in the *IFN-γ* gene, mainly rs2069705 (−1615 C/T) and rs2430561 (+874 T/A), with the risk of breast cancer [29], [44]–[47], but these results also conflicted with null, positive, and inverse associations. In the present study, we found a null association of *IFN-γ* rs2069705 with the risk of breast cancer among the study participants as a whole. However, we observed a positive association among the women with <1 fever per year but an inverse association among women with ≥1 fever per year for the CT/TT genotypes and breast cancer risk. These phenomena may explain the previously mentioned inconsistent results to some extent, and suggest that IFN-γ plays a role in tumorigenesis depending on certain environments, such as febrile infections.

The evidence of interplay between fevers and IFN-γ has been noted in previous experimental studies that show how fever can induce IFN-γ and be induced by IFN-γ [14]–[16], [19]–[22]. Moreover, it has been shown that a low level of IFN-γ promotes tumor development and a high level of IFN-γ mediates significant antitumor effects [25]. Based on the evidence that the T allele is associated with a higher level of IFN-γ than the C allele [27], [28], [48], the co-existence of a fever and the T allele of *IFN-γ* rs2069705 might increase the IFN-γ level high enough to make the host's anticancer defense more effective while the co-existence of less fever and the T allele might keep IFN-γ at a certain low level to promote tumourgenesis, supporting our results to some extent that women with CT/TT genotypes and ≥1 fever per year have a decreased breast cancer risk and women with CT/TT genotypes and <1 fever per year have an increased breast cancer risk. Nevertheless, the exact mechanisms in which fever and IFN-γ interact on breast cancer risk remain to be studied.

Several potential limitations of this study should be mentioned. In a case-control study, it is unrealistic to blind disease status for interviewers and the study is subject to unconscious interviewer bias. We have attempted to reduce this potential bias source as much as possible by ensuring that the interviewers were unaware of this study's point of interest. Recall bias is another unavoidable concern in a case-control study. However, misclassification due to recall bias occurred equally in both groups of patients and controls. This non-differential misclassification meets the conditions to reduce test power and bias study estimators toward no association [49]. In the present study, moreover, due to an awareness of their disease status, patients might be more likely to recall their fever histories than controls, resulting in a more underestimated association. Therefore, the positive association between febrile infections and breast cancer risk cannot be completely explained by biased information. All of the participants in the present study were Han Chinese. Therefore, the results were not affected by population admixture or genetic heterogeneity. In addition, the frequency of the *IFN-γ* rs2069705 C allele among the healthy controls was 0.73 in our study, which was similar to that found in other populations within Asia (0.75 in a Taiwanese population and 0.81 in another Korean population [28], [50].

In summary, our findings indicate a possible link between fever history and a decreased risk of breast cancer for the first time, providing novel information for prevention of breast cancer, or even other cancers, in line with previous reports on the association between infections and melanoma risk conducted by Kolmel KF et al [6], [38]. Moreover, we find that the association of fever frequency with the risk of breast cancer was significantly modified by a genetic polymorphism of *IFN-γ* rs2069705 and that the association of the polymorphism with the risk of breast cancer was significantly modified by previous fever frequency, which may provide new insight into hyperthermic treatment and interferon therapy. The possible antitumor effects of fever, as shown in the present study, also have important implications for the prudent clinical and public use of antibiotics and antipyretics.

## Materials and Methods

### Study population

Female patients, newly histologically diagnosed with breast cancer between October 2008 and June 2010 in the First and Second Affiliated Hospitals and the Cancer Center of Sun Yat-sen University in Guangzhou, China, were consecutively recruited to this study. The study methods are described elsewhere [51]. A total of 839 eligible breast cancer cases during the study period completed in-person interviews with response rates of 75% to 85% depending on the hospitals. Age (within 5 years) frequency-matched female controls were identified from the primary care databases of the same hospitals as breast cancer cases during the same period. Women who self-reported a history of cancer were excluded. Of the eligible controls, 863 (78.2%) completed in-person interviews. All subjects must have resided in the Guangzhou area for at least 5 years. Written informed consent was obtained for the interviews and the specimen collections from each subject. The Ethical Committee of the School of Public Health at Sun Yat-sen University approved the study.

### Data collection and laboratory protocol

Cases and controls were interviewed face-to-face by trained interviewers using the same questionnaire. The following information was obtained from the interviews: menstrual and reproductive history, personal history of diseases including febrile acute infections, family history of cancer, height and weight, and demographic factors. In the instance of infections, subjects were asked to recall the average number of times per year during the last 10 years that they had experienced a fever or body temperature ≥38°C as the result of acute febrile infections including influenza, common cold, infectious enteritis, bronchitis, pneumonia, and herpes simplex (<1, 1∼2, 3∼4, or ≥5 times). Information about these infections was successfully collected from 731 cases (87.1% of those eligible) and 777 controls (90.0% of those eligible). The interviewers did not know the hypotheses of this study.

Blood samples were collected immediately after admission to the hospital for patients or after the interview for controls and were stored at −80°C. The clinical characteristics of the breast cancer patients were collected from medical records. The statuses of oestrogen receptor (ER), progesterone receptor (PR), and human epidermal growth factor receptor 2 (HER2) for the breast cancer were determined by pathologists using immunohistochemistry tests. The definitions of statuses of ER, PR and HER2 statuses were described in detail in our previous study [52].

Genomic DNA was extracted from the buffy coats of the participants using the TIANamp Genomic DNA Kit (TianGen Biotech Co., Ltd., Beijing, China) and genotyped using a matrix-assisted laser desorption/ionization time-of-flight (MALDI-TOF) mass spectrometry platform (Sequenom, San Diego, California, USA), according to the manufacturer's instructions. The details of the primers are described elsewhere [51]. Duplicate samples (5% of the total) were included for the evaluation of genotyping quality and the concordance rate was 100%. Genotyping was successfully performed among 807 (96.2%) cases and 840 (97.3%) controls for *IFN-γ* rs2069705. No deviation from the Hardy-Weinberg equilibrium was observed (P = 0.350 in control group).

### Statistical analysis

The differences in demographic characteristics and common risk factors for breast cancer between the cases and controls were tested using the χ^2^ test (for categorical variables) or Student's t-test (for continuous variables). Differences in fever frequency between cases and controls were tested using the Mann-Whitney U test. The Hardy–Weinberg equilibrium for *IFN-γ* rs2069705 was evaluated using a goodness-of-fit χ^2^ test to compare the observed genotype frequency with the expected one among the controls. Multivariate logistic regression models were used to assess the effects of fever history and genotype on breast cancer risk, controlling for age and for the potential risk factors of breast cancer (age, BMI, age at menarche, marital status, education, parity, menopausal status, and family history of breast cancer), which were defined categorically with the exception of age (Table 1). Models were fit using fever frequencies as categorical variable. For evaluating the dose-response association between fever frequency and the risk of breast cancer, tests for trends were performed by entering the categorical variables as continuous variables in the model. Stratified analyses for the associations between fever frequency, genotype, and the risk of breast cancer were performed by menopausal status, BMI, and clinical characteristics. The heterogeneity of the odds ratios between the different clinical characteristics levels was assessed using a multivariable logistic regression model restricted to cases (case-only analysis) with the clinical characteristics as the outcome variables and adjusting for potential breast cancer risk factors. We assumed a dominant inheritance for *IFN-γ* rs2069705 and the mutant homozygotes and heterozygotes were combined because the effects' directions of the CT and TT genotypes were the same (OR>1).

The interaction between fever frequency and genotype on breast cancer risk was evaluated by multiplicative and additive models. We tested for multiplicative interaction by including the product term in multivariate logistic regression. Additive interaction was assessed using a method proposed by Rothman [53], [54]. All statistical tests were two-tailed with P<0.05 considered to be significant. Statistical analyses were performed using SAS 9.2 (SAS Institute Inc., Cary, NC, USA).
